# Phosphorylation of Serine 225 in Hepatitis C Virus NS5A Regulates Protein-Protein Interactions

**DOI:** 10.1128/JVI.00805-17

**Published:** 2017-08-10

**Authors:** Niluka Goonawardane, Anna Gebhardt, Christopher Bartlett, Andreas Pichlmair, Mark Harris

**Affiliations:** a School of Molecular and Cellular Biology, Faculty of Biological Sciences and Astbury Centre for Structural Molecular Biology, University of Leeds, Leeds, United Kingdom; b Max Planck Institute of Biochemistry, Martinsried, Germany; Washington University School of Medicine

**Keywords:** hepatitis C virus, NS5A, phosphorylation, RNA replication, protein phosphorylation, subgenomic replicon

## Abstract

Hepatitis C virus (HCV) nonstructural protein 5A (NS5A) is a phosphoprotein that plays key, yet poorly defined, roles in both virus genome replication and virion assembly/release. It has been proposed that differential phosphorylation could act as a switch to regulate the various functions of NS5A; however, the mechanistic details of the role of this posttranslational modification in the virus life cycle remain obscure. We previously reported (D. Ross-Thriepland, J. Mankouri, and M. Harris, J Virol 89:3123–3135, 2015, doi:10.1128/JVI.02995-14) a role for phosphorylation at serine 225 (S225) of NS5A in the regulation of JFH-1 (genotype 2a) genome replication. A phosphoablatant (S225A) mutation resulted in a 10-fold reduction in replication and a perinuclear restricted distribution of NS5A, whereas the corresponding phosphomimetic mutation (S225D) had no phenotype. To determine the molecular mechanisms underpinning this phenotype we conducted a label-free proteomics approach to identify cellular NS5A interaction partners. This analysis revealed that the S225A mutation disrupted the interactions of NS5A with a number of cellular proteins, in particular the nucleosome assembly protein 1-like protein 1 (NAP1L1), bridging integrator 1 (Bin1, also known as amphiphysin II), and vesicle-associated membrane protein-associated protein A (VAP-A). These interactions were validated by immunoprecipitation/Western blotting, immunofluorescence, and proximity ligation assay. Importantly, small interfering RNA (siRNA)-mediated knockdown of NAP1L1, Bin1 or VAP-A impaired viral genome replication and recapitulated the perinuclear redistribution of NS5A seen in the S225A mutant. These results demonstrate that S225 phosphorylation regulates the interactions of NS5A with a defined subset of cellular proteins. Furthermore, these interactions regulate both HCV genome replication and the subcellular localization of replication complexes.

**IMPORTANCE** Hepatitis C virus is an important human pathogen. The viral nonstructural 5A protein (NS5A) is the target for new antiviral drugs. NS5A has multiple functions during the virus life cycle, but the biochemical details of these roles remain obscure. NS5A is known to be phosphorylated by cellular protein kinases, and in this study, we set out to determine whether this modification is required for the binding of NS5A to other cellular proteins. We identified 3 such proteins and show that they interacted only with NS5A that was phosphorylated on a specific residue. Furthermore, these proteins were required for efficient virus replication and the ability of NS5A to spread throughout the cytoplasm of the cell. Our results help to define the function of NS5A and may contribute to an understanding of the mode of action of the highly potent antiviral drugs that are targeted to NS5A.

## INTRODUCTION

Hepatitis C virus (HCV) infects approximately 130 to 170 million individuals worldwide and is a leading cause of liver disease (1). There is no vaccine available, and current antiviral treatments are less effective against some viral strains (2). HCV belongs to the Flaviviridae family (genus Hepacivirus) of enveloped viruses with a positive-sense RNA genome (9.6 kb) coding for a single polyprotein that is processed co- and posttranslationally by viral and host proteases, yielding four structural proteins (core, E1, E2, and p7) and six nonstructural proteins (NS2, NS3, NS4A, NS4B, NS5A, and NS5B) (3). NS3 to NS5B are necessary and sufficient for viral genome replication (4) and thus constitute the essential components of the genome replication complex. Further to its requirement in genome replication, NS5A has been shown to play a critical role in virion assembly, as discussed below.

Early in infection, HCV remodels endoplasmic reticulum (ER)-derived membranes to form a “membranous web” (MW) comprised of single, double, and multimembrane vesicles (SMVs, DMVs, and MMVs) that are enriched in viral (e.g., NS3, NS5A, and NS5B) and host cell proteins (5, 6). The MW is proposed as the site of viral genome replication, and in Huh7 cells, the MW is extensively distributed throughout the cytoplasm, correlating with the observed subcellular distribution of NS5A as discrete punctae throughout the cytoplasm. The cellular lipid kinase, phosphatidylinositol kinase type III alpha isoform (PI4KIIIα), is activated by NS5A (7), and the subsequent increase in abundance of phosphatidylinositol-4-phosphate (PI4P) is critical for establishment and maintenance of the MW (8–10). NS5A is also thought to be involved in delivery of nascent virus genomes from the MW to sites of assembly. While the latter are yet to be unambiguously defined, it is accepted that an association of both NS5A and the HCV capsid (core) protein with lipid droplets (LDs; a host organelle responsible for storage of neutral lipids) is required during this process. It has been hypothesized that NS5A switches from a role in replication to an alternative function in assembly which might involve transporting nascent genomes via LDs to assembly sites.

**FIG 1:**
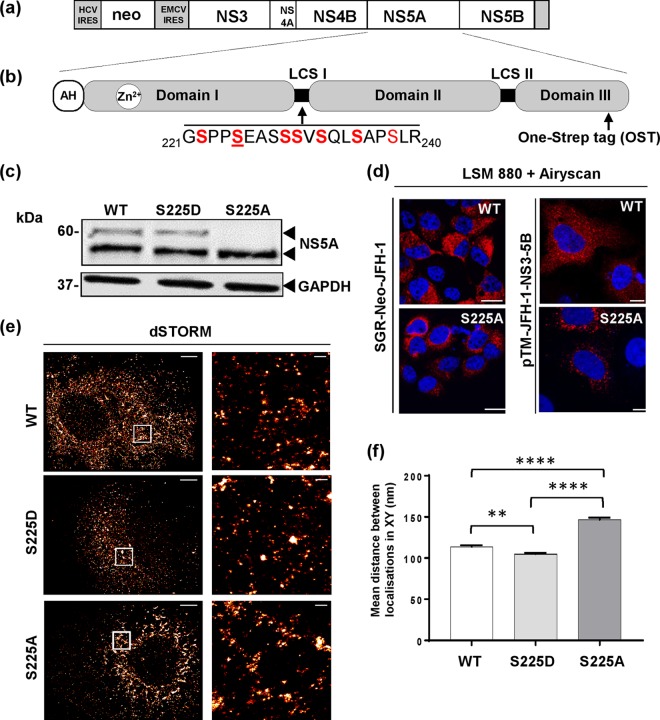
Construction and validation of One-Strep-tagged (OST) NS5A neomycin reporter SGR JFH-1. (a) Schematic of SGR-Neo-JFH-1-5A-OST. (b) NS5A structure showing amino acid residues for LCSI and location of the OST. The OST was introduced between residues 418 and 419, a site previously shown to tolerate insertions (25). AH, amphipathic helix; neo, neomycin phosphotransferase. (c) G418-resistant Huh7 cells stably harboring the SGRs were isolated by selective culture. Cells were lysed and analyzed by Western blotting for NS5A or glyceraldehyde-3-phosphate dehydrogenase (GAPDH). (d) SGR-harboring Huh7 cells (left) or Huh7-Lunet T7 cells transfected with pTM-NS3-5B plasmids (right) were seeded onto coverslips, fixed at 48 h posttransfection (hpt), and permeabilized prior to staining with a sheep polyclonal anti-NS5A serum and Alexa Fluor 594 secondary antibody. Scale bars, 30 μm and 20 μm. (e) Huh7 cells stably harboring wild-type, S225D, or S225A SGRs were fixed, immunostained for NS5A (Alexa Fluor 647-labeled 9E10), and imaged by dSTORM. Images were reconstructed from 873185, 354952, and 641609 localizations for wild-type, S225D, and S225A SGRs, respectively. Histogram bins of 100 nm and 10 nm (with image smoothing) were used for the left and right images, respectively. Scale bars, 5 μm (left) and 500 nm (right). (f) Size of NS5A protein clusters identified by DBSCAN analysis. The mean Euclidean distance between each localization to every other localization in an identified cluster was measured. Data represent the means ± SEM from three independent cells containing 2,062, 1,561, and 1,515 clusters from wild-type, S225D, and S225A SGRs, respectively. Significant differences are indicated as follows: **, *P* < 0.005, and ****, *P* < 0.0001.

In this regard, NS5A is highly phosphorylated, and it is possible that this reversible posttranslational modification could mediate a switch in NS5A function, by altering protein conformation and/or protein-protein interactions. NS5A comprises three domains and is tethered to membranes by an N-terminal amphipathic helix (Fig. 1b). Domain I is highly structured (11–13), while domains II and III are intrinsically disordered, with elements of transient secondary structure (14). The domains are linked by low-complexity sequences (LCS); LCSI is serine rich, and LCSII is proline rich. To address the potential functional role of NS5A phosphorylation, we and others have used mass spectrometry (MS) to identify phosphorylation sites (15–20). These studies have identified multiple phosphorylation sites, but in particular they show that LCSI is highly phosphorylated. Subsequent mutagenesis of these phosphorylation sites revealed that a subset of them is required for efficient genome replication. In particular, a number of groups have presented evidence that phosphorylation of serine 235 is critical, as replacement of this residue with alanine (S235A) resulted in a 100-fold reduction in genome replication (15–17, 21). It should be noted that other approaches, such as *in vitro* phosphorylation assays, genetic approaches, and use of selective inhibitors, have identified additional sites toward the C terminus of NS5A, such as T360 (22) and S457 (23).

In this study, we focused on another phosphorylated residue within LCSI: serine 225. We demonstrated previously that phosphorylation of serine 225 was required for efficient genome replication and contributed to the subcellular localization of viral proteins during infection (19, 24). Alanine substitution (S225A) resulted in a 10-fold reduction in genome replication and was concomitant with a restricted distribution of NS5A, and other factors known to participate in genome replication (NS3 and PI4P lipids), to a perinuclear region (24). This restriction was dramatic compared to the extensive distribution of these components throughout the cytoplasm in wild-type (WT)-infected cells.

To understand the molecular mechanism underpinning the phenotype of the S225A mutation, we hypothesized that it might be explained by a role of S225 phosphorylation in regulating interactions between NS5A and cellular proteins. To address this, we used affinity purification of One-Strep-tagged NS5A in conjunction with label-free quantitative proteomics analysis to compare the interactome of wild-type and S225 mutants of NS5A. In this study, we focused on 3 cellular proteins—nucleosome assembly protein 1-like protein 1 (NAP1L1), bridging integrator 1 (Bin1, also known as amphiphysin II), and vesicle-associated membrane protein-associated protein A (VAP-A)—that exhibited a loss of interaction with S225A mutant NS5A compared to the wild type and the S225D mutant. In contrast, the binding of VAP-B to NS5A was not dependent on S225 phosphorylation and acted as a control. These interactions were validated by immunoprecipitation, immunofluorescence, and proximity ligation assay (PLA). Furthermore, small interfering RNA (siRNA) ablation of endogenous NAP1L1, Bin1, and VAP-A reduced both HCV RNA replication and NS5A expression significantly; however, consistent with lack of dependence on S225 phosphorylation, VAP-B ablation had a modest effect. Importantly, ablation of NAP1L1, Bin1, and VAP-A recapitulated the restricted distribution of the NS5A protein. We propose that S225 phosphorylation is required for the interaction of NS5A with these cellular proteins and enables the formation and distribution of replication complexes throughout the cytoplasm, thereby enhancing the efficiency of genome replication.

## RESULTS

### Identification of serine 225 phosphorylation-dependent NS5A-interacting proteins.

Previously, we reported that phosphorylation of serine 225 within LCSI of NS5A played a role in the regulation of JFH-1 genome replication (24). Mutation of this residue to alanine (S225A; phosphoablatant) resulted in a 10-fold reduction in genome replication and altered subcellular distribution of NS5A, whereas the phosphomimetic mutation (S225D) had no phenotype. To understand the mechanism behind this phenotype, we sought to identify cellular proteins that interacted with NS5A in an S225 phosphorylation-dependent fashion. For this, we exploited the One-Strep tag (OST) affinity purification strategy that we had previously used to identify sites of phosphorylation within NS5A (19). The OST is a peptide that structurally resembles biotin and binds to recombinant streptavidin (Strep-Tactin). S225A and S225D mutations were cloned into pSGR-Neo-JFH-1-5A-OST, which contained the OST cloned into a well-tolerated insertion site near the C terminus of NS5A domain III (25) (Fig. 1a and b). These subgenomic replicons (SGRs) were used to establish stable Huh7 cell lines expressing either the wild-type or the two mutant SGRs. The phenotype of these mutants was confirmed by Western blotting and fluorescence microscopy (Fig. 1c and d). As expected (24), the S225A mutation resulted in a reduction in hyperphosphorylation (Fig. 1c) and in a distribution of the protein that was restricted to the perinuclear region (Fig. 1d). We had previously demonstrated that this phenotype was not a consequence of the reduced level of RNA replication exhibited by the S225A mutant (24). We further confirmed this by expressing either wild-type or S225A mutant NS5A in the context of the NS3-NS5B polyprotein from a T7 RNA polymerase-driven construct (pTM, a kind gift from Volker Lohmann). Following transfection of these plasmids into Huh7-Lunet T7 cells (stably expressing T7 RNA polymerase, also a kind gift from Volker Lohmann), the restricted distribution of NS5A S225A was recapitulated (Fig. 1d), confirming that the phenotype was not dependent on genome replication or the level of NS5A expression. We also applied a superresolution approach (Fig. 1e and f), and this revealed an additional S225A phenotype: discrete clusters of NS5A localizations were equivalent to the diffraction limited puncta observed by wide-field microscopy. Clusters were observed distributed throughout the cytoplasm apart from the S225A mutant, which was more condensed and perinuclear. Consistent with our previous findings (24), larger NS5A clusters were observed for the S225A mutant than for the wild type and the S225D mutant. Taken together, these data demonstrate that S225 phosphorylation regulated not only the distribution of the replication complexes but also their architecture. These observations gave further impetus for the need to understand the molecular mechanism behind this phenotype.

**FIG 2:**
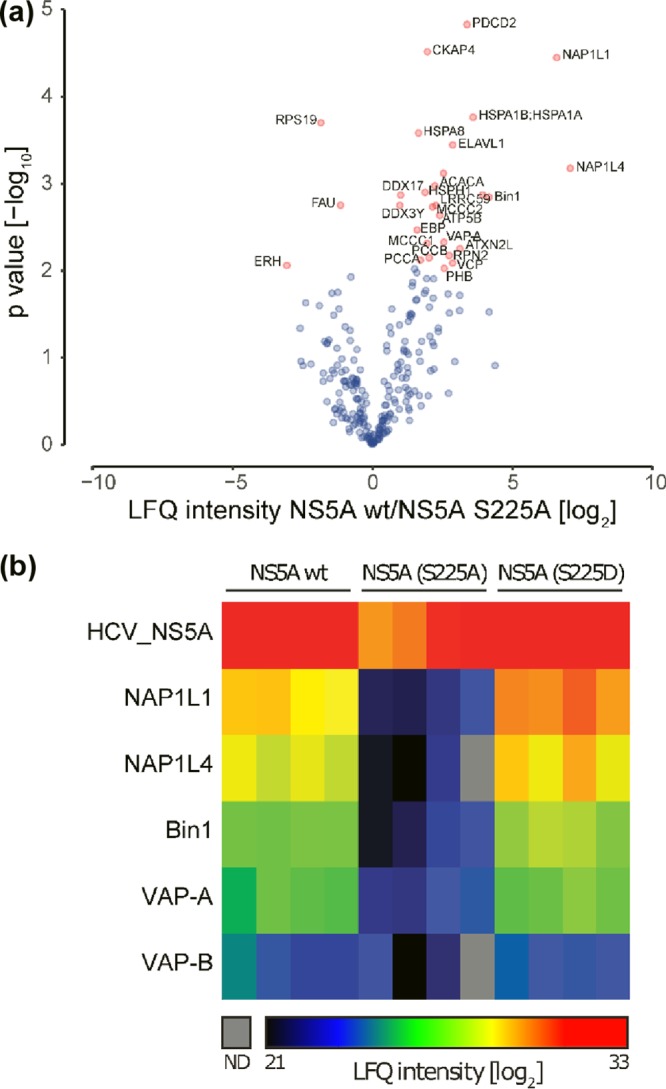
Proteomic analysis of NS5A-interacting proteins. OST-tagged wild-type (wt), S225A, and S225D NS5A proteins were affinity purified from Huh7 cells stably harboring SGRs, and associated proteins were identified by LC-MS/MS. Four independent affinity purifications were performed for each bait. (a) Volcano plot displaying the average degree of enrichment by wt NS5A over S225A NS5A (ratio of label-free quantification [LFQ] protein intensities) and the *P* value (Welch's *t* test) for each protein. Significantly enriched cellular proteins are highlighted in red. (b) Heat map showing nonimputed log_2_ transformed LFQ intensities for each individual replicate in rainbow colors (see color scale). Only the bait protein and selected cellular interaction partners are depicted in the plot. Gray color represents missing values (not determined [ND]).

To identify cellular candidates that are potentially involved in the S225A phenotype, we next performed affinity purification from cytoplasmic lysates of Strep-tagged wild-type, S225A, and S225D NS5A and analyzed the bound fractions by mass spectrometry. A large number of known NS5A binding proteins were identified by this procedure. Wild-type NS5A and S225D mutant NS5A showed no difference in their binding to associated proteins (see Data Set S1 in the supplemental material). As we were interested in the role of S225 phosphorylation, we mined the data set for those cellular proteins that bound well to wild-type or S225D NS5A but exhibited a reduced level of interaction with S225A mutant NS5A (Fig. 2). We focused on three of these differentially enriched proteins. Bin1 (also known as amphiphysin II) is a BAR domain-containing protein involved in generating membrane curvature and was previously characterized as an NS5A interactor (26–28). VAP-A (also known as hVAP-33) is a vesicle-associated protein previously shown to participate in the formation of HCV genome replication complexes and to bind to NS5A (29–32). As well as these previously characterized NS5A partners, we also focused on the nucleosome assembly protein 1-like proteins 1 and 4 (NAP1L1 and NAP1L4). NAP1L1 has been shown to interact with HCV core and NS5A (33, 34) as well as Kaposi's sarcoma herpesvirus (KSHV) LANA (35) and HIV-1 Tat (36). Although its roles in the nucleus associated with chromatin structure and gene expression are well defined, NAP1L1 is predominantly located in the cytoplasm, where its function(s) remains to be elucidated. We chose these proteins for further analysis because they exhibited the greatest difference between wild-type and S225A NS5A (Fig. 2b) and thus represented the best candidates to explain the mechanism underpinning the S225A phenotype. As a control, we also examined VAP-B, which was enriched as an NS5A interactor but did not exhibit S225 phosphorylation dependence.

### Validation of S225 phosphorylation-dependent interacting proteins.

**FIG 3:**
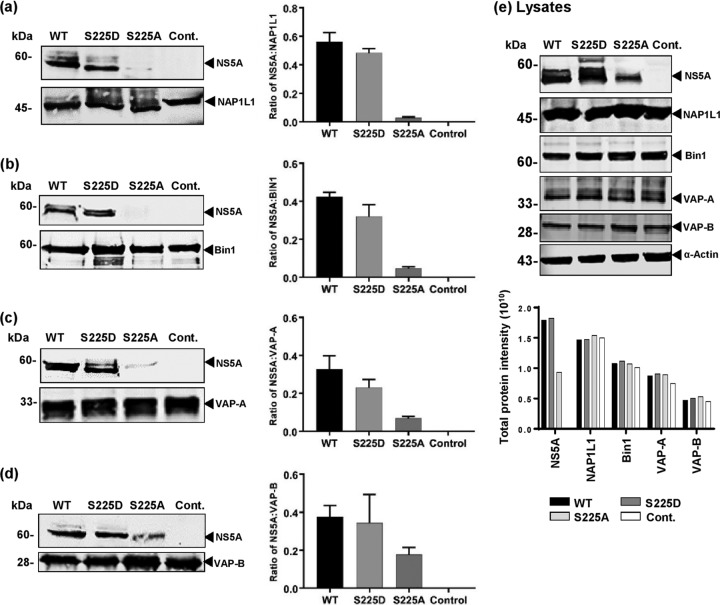
Validation of NS5A-interacting proteins NAP1L1, Bin1, VAP-A, and VAP-B. Huh7 cells stably harboring wild-type (WT), S225D, or S225A SGRs were lysed and immunoprecipitated with antibodies to NAP1L1 (a), Bin1 (b), VAP-A (c), and VAP-B (d) prior to analysis by Western blotting with mouse monoclonal antiserum to NS5A or antibodies to the indicated cellular proteins. The ratios of NS5A to the cellular proteins were quantified from fluorescent Western blots (*n* = 3). Western blots are representative of three independent experiments. (e) Levels of NS5A and cellular proteins in cytoplasmic lysates were confirmed by Western blotting and quantified (graph). Cont., control.

We next sought to validate the proteomic analysis/mass spectrometry results by a specific immunoprecipitation approach. To this end, lysates from stable Huh7 cell lines harboring either the wild-type or S225A/S225D mutant SGRs, or control parental cells, were immunoprecipitated with antibodies to NAP1L1, Bin1, VAP-A, or VAP-B. The immunoprecipitates were probed with an anti-NS5A antiserum by Western blotting (Fig. 3a to d). The adjacent graphs show quantification of 3 independent assays. Consistent with the proteomic analysis, wild-type and S225D mutant NS5A bound equally to all four cellular proteins; in contrast, S225A NS5A bound weakly, or not at all, to NAP1L1, Bin1, and VAP-A. In addition, binding to VAP-B was confirmed to be less dependent on S225 phosphorylation, as there was only a 50% reduction in the level of S225A NS5A bound to VAP-B. Input levels of NS5A and the four cellular proteins were confirmed by Western blotting of lysates and quantification (Fig. 3e, graph). As seen previously, levels of S225A NS5A were reduced by approximately 50%; however, this did not account for the reduction in levels of NS5A bound to NAP1L1, Bin1, or VAP-A. As the reduction in expression of S225A correlated with the observed reduction in the amount of NS5A coimmunoprecipitated with VAP-B, we confirm that the NS5A–VAP-B interaction was not dependent on S225 phosphorylation.

In order to further validate the interactions between NS5A and either NAP1L1 or Bin1, we applied two imaging techniques to examine their colocalization and interaction in Huh7 cells stably harboring SGRs. To complement conventional coimmunofluorescence, which can only determine colocalization, we used the proximity ligation assay (PLA), which allows the detection of direct protein-protein interactions in intact cells (37) PLA involves the use of oligonucleotides attached to secondary antibodies which guide the formation of circular DNA strands when bound in close proximity (approximately 5 to 30 nm). These DNA circles then template localized rolling-circle amplification (RCA), allowing individual interacting pairs of proteins to be visualized and enumerated in fixed samples (37, 38). We were able to apply the PLA technique only for NAP1L1 and Bin1, due to a lack of suitable antibody pairs for VAP-A and VAP-B; thus, we also restricted the coimmunofluorescence analysis to these two proteins.

**FIG 4:**
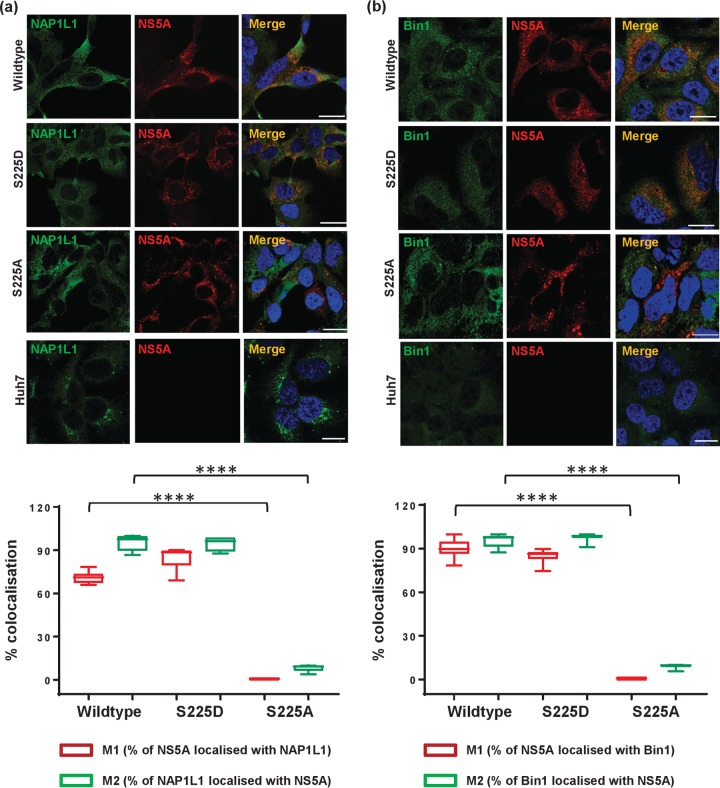
Colocalization of NS5A S225 mutants with cellular proteins. Parental Huh7 cells or cells stably harboring wild-type (WT), S225D, or S225A SGRs were seeded onto coverslips and incubated for 72 h prior to fixation. Cells were permeabilized and immunostained for NS5A (red) and either NAP1L1 (a, green), or Bin1 (b, green). Scale bar, 20 μm. Box-and-whisker plots show the percent colocalization between NS5A and either NAP1L1 or Bin1. Manders' overlap coefficients M1 and M2 were used to evaluate the degree of colocalization between two fluorescent labels (red and green) in 10 cells. ****, *P* < 0.0001.

**FIG 5:**
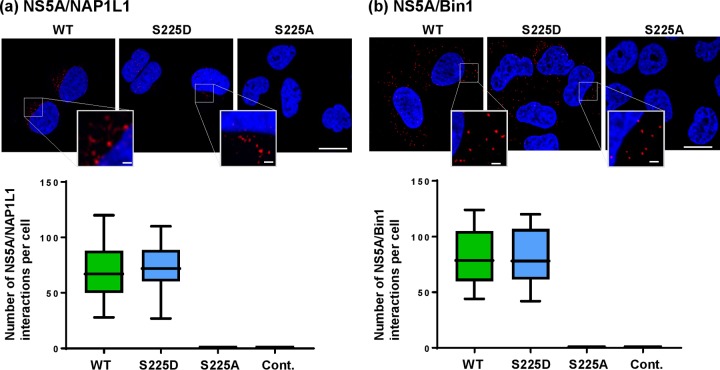
Proximity ligation assay for NS5A interactions with NAP1L1 and Bin1. Huh7 cells stably harboring wild-type, S225D, or S225A SGRs were seeded onto coverslips and incubated for 72 h prior to fixation and immunostaining for NS5A and either NAP1L1 or Bin1, followed by PLA probe MINUS and PLUS (Sigma-Aldrich) ligation and rolling-circle amplification (RCA). The hybridization probes were labeled with Alexa Fluor 555 (red), and the nuclei were counterstained with DAPI (blue). Scale bars, 20 μm and 2 μm. For each experiment, the PLA signals (per cell) of each sample were counted using Imaris software version 7.4 (Bitplane AG) from a minimum of 20 cells.

Both NAP1L1 (Fig. 4a) and Bin1 (Fig. 4b) were distributed diffusely throughout the cytoplasm; in the context of both wild-type and S225D NS5A SGR, there was extensive colocalization with NS5A which was particularly noticeable close to the nucleus and not so apparent at the periphery of the cell (quantification shown in lower part of Fig. 4). S225A NS5A showed a different picture: NS5A and either NAP1L1 or Bin1 seemed to occupy mutually exclusive areas within the cytoplasm, and indeed, quantification showed no significant colocalization. As shown in Fig. 5, we observed strong fluorescence signals (red punctae) for both NS5A/NAP1L1 (Fig. 5a) and NS5A/Bin1 (Fig. 5b) in the cytoplasm of Huh7 cells harboring either wild-type or S225D SGR. In contrast, no PLA signal was observed for either NAP1L1 or Bin1 in cells harboring the S225A mutant SGR. These data confirm that NS5A interacts with both NAP1L1 and Bin1 in the cytoplasm of SGR-harboring cells in an S225 phosphorylation-dependent fashion.

### S225 phosphorylation-dependent NS5A-interacting proteins are required for efficient viral genome replication.

**FIG 6:**
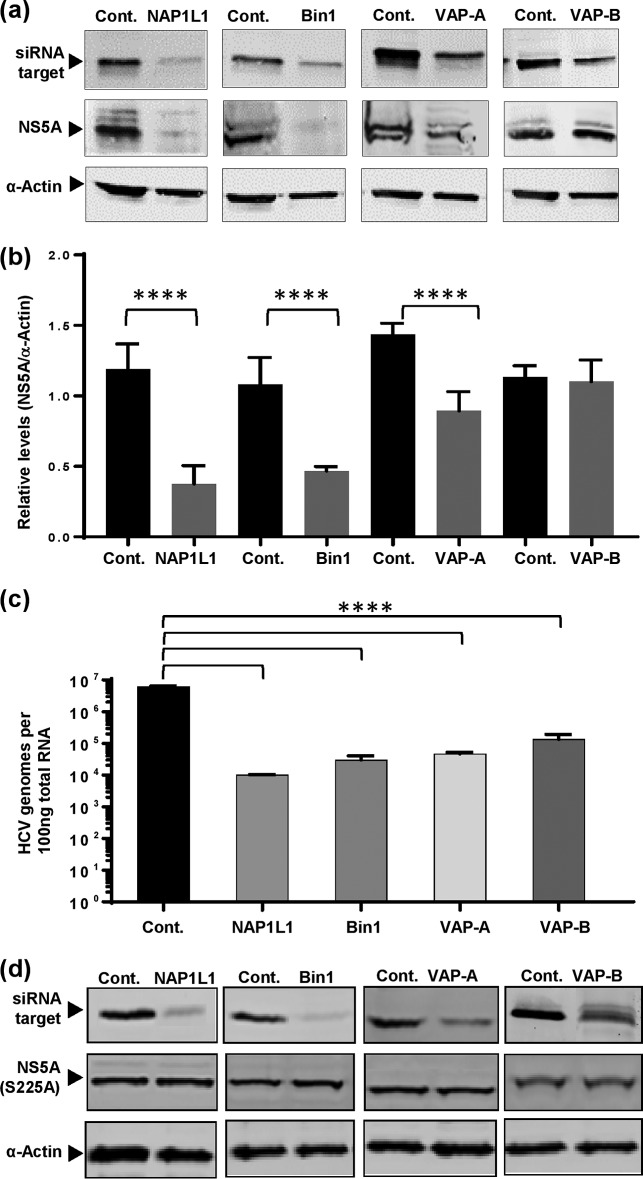
Effects of siRNA ablation of NS5A-interacting protein expression on NS5A expression and RNA replication. (a) Huh7 cells stably harboring a wild-type SGR were transfected with pooled siRNA targeting NAP1L1, Bin1, VAP-A, or VAP-B or a control scrambled siRNA (Santa Cruz) at a final concentration of 10 nM. Transfected cells were incubated in medium lacking G418, lysed at 72 hpt, and analyzed by Western blotting. (b) Total NS5A levels were quantified from fluorescent Western blots (*n* = 3). The Western blots are representative of three independent experiments. ****, *P* < 0.0001 compared to the value for the control. (c) At 72 hpt, the cells were harvested in TRIzol, total RNA was extracted, and qRT-PCR was conducted on 100 ng of total cellular RNA using 5′-UTR TaqMan primers (53). The data are from three independent experiments. (d) Huh7 cells stably harboring the S225A mutant SGR were transfected with indicated siRNA and analyzed by Western blotting at 72 hpt as described for panel a.

We previously demonstrated that the S225A mutation in the context of either an SGR or infectious virus resulted in a 1-log reduction in genome replication (19, 24). Because this mutation also disrupted binding of NS5A to NAP1L1, Bin1, VAP-A, and, to a lesser extent, VAP-B, we hypothesized that these cellular proteins might play a role in genome replication. To test this, we adopted an siRNA approach to ablate expression of each of these cellular proteins in Huh7 cells stably harboring a wild-type JFH-1 SGR. Cells were transfected with siRNA pools targeting different sites of NAP1L1, Bin1, VAP-A, or VAP-B or with a control nontargeting siRNA and harvested at 72 hours posttransfection (hpt), and protein expression levels were determined by Western blot analysis. As shown in Fig. 6a, ablation of NAP1L1 and Bin1 expression was efficient and resulted in a concomitant reduction in NS5A protein levels (Fig. 6b). The siRNAs for VAP-A and VAP-B were less efficient; however, VAP-A ablation did reduce NS5A expression, although not as effectively as NAP1L1 or Bin1. In contrast, we observed no significant effect of the VAP-B ablation on NS5A levels, possibly because, as reported previously, the role of VAP-B in HCV genome replication is mediated via interactions with both NS5B and NS5A (39). As NS5A expression levels are an indirect measure of genome replication, we also directly assessed the levels of SGR RNA in siRNA-transfected cells by real-time quantitative PCR (qRT-PCR) (Fig. 6c). Consistent with the effects of NAP1L1, Bin1, and VAP-A ablation, we observed reductions of between 100- and 1,000-fold in HCV-specific RNA levels compared to the levels in cells transfected with the control siRNA. Interestingly, despite the fact that VAP-B ablation had no effect on NS5A levels (Fig. 6b), it also significantly reduced HCV RNA levels, although not as effectively as the other 3 targets. Lastly, we confirmed that the ablation of NAP1L1, Bin1, VAP-A, or VAP-B expression had no effect on levels of S225A NS5A (Fig. 6d). These data confirm that the S225 phosphorylation-dependent interacting proteins NAP1L1, Bin1, and VAP-A (and, to a lesser extent, VAP-B) are involved in HCV genome replication.

### The subcellular distribution of NS5A is regulated by S225 phosphorylation-dependent binding to NAP1L1, Bin1, and VAP-A.

**FIG 7:**
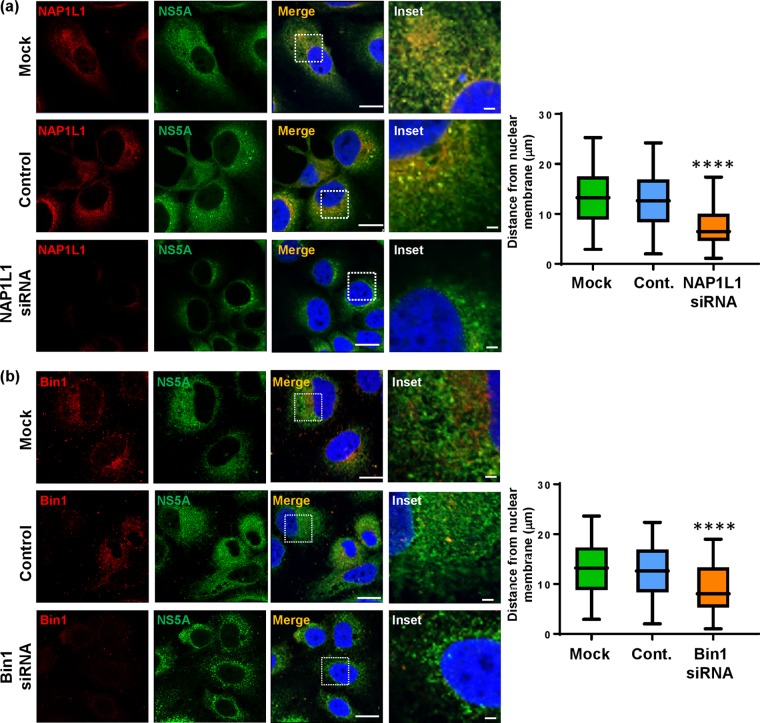
Effects of siRNA ablation of NAP1L1 or Bin1 on the distribution of NS5A. Huh7 cells stably harboring a wild-type SGR were left untransfected or were transfected with pooled siRNA targeting NAP1L1 or Bin1 or a control scrambled siRNA (Santa Cruz) at a final concentration of 10 nM. At 72 hpt, cells were permeabilized, immunostained for NS5A (green), NAP1L1 (a, red), or Bin1 (b, red), and imaged by confocal microscopy. Scale bars, 20 μm and 5 μm. Spatial data for NS5A were determined from 12 cells for each assay using the ImageJ software package. The data are representative of three independent experiments. ****, *P* < 0.0001 compared to the control value.

**FIG 8:**
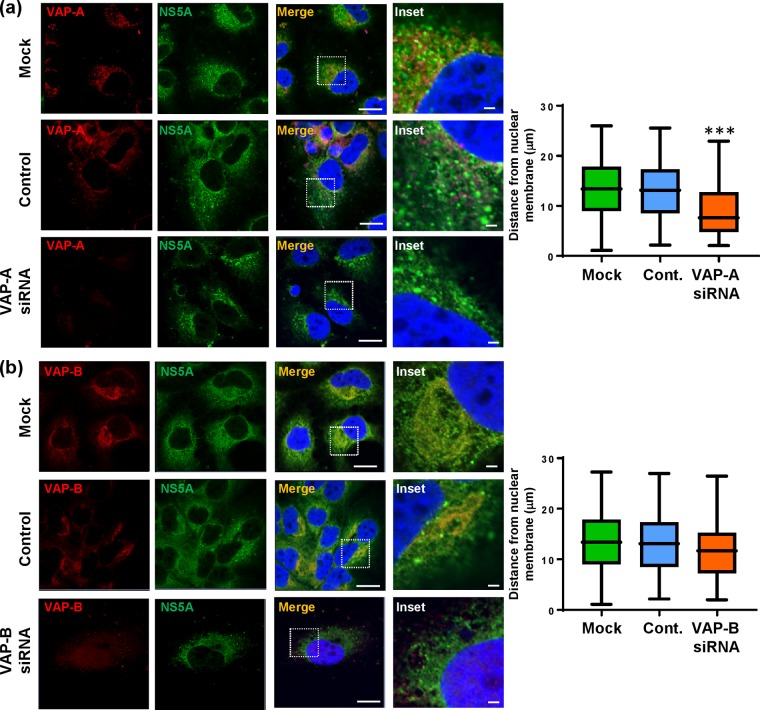
Effects of siRNA ablation of VAP-A or VAP-B on the distribution of NS5A. Huh7 cells stably harboring a wild-type SGR were left untransfected or were transfected with pooled siRNA targeting VAP-A or VAP-B or a control scrambled siRNA (Santa Cruz) at a final concentration of 10 nM. At 72 hpt, cells were permeabilized, immunostained for NS5A (green), VAP-A (a, red), or VAP-B (b, red) and imaged by confocal microscopy. Scale bars, 20 μm and 5 μm. Spatial data for NS5A were determined from 12 cells for each assay using the ImageJ software package. The data are representative of those from three independent experiments. ***, *P* < 0.001 compared to the control value.

**FIG 9:**
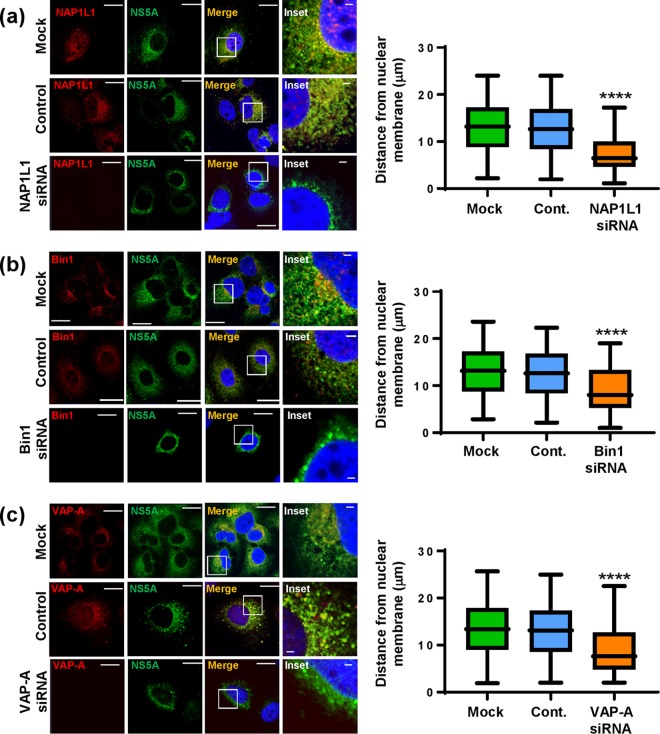
Effects of siRNA ablation of NAP1L1, Bin1, or VAP-A on the distribution of NS5A in the absence of genome replication. Huh7-Lunet T7 cells were left untransfected or were transfected with pooled siRNA targeting NAP1L1, Bin1, or VAP-A or a control scrambled siRNA (Santa Cruz) at a final concentration of 10 nM. At 48 hpt, cells were transfected with pTM-NS3-5B plasmid. After a further 24 h, cells were permeabilized, immunostained for NS5A (green), NAP1L1 (a, red), Bin1 (b, red), or VAP-A (c, red), and imaged by confocal microscopy. Scale bars, 20 μm and 5 μm. Spatial data for NS5A were determined from 20 cells for each assay using the ImageJ software package. ****, *P* < 0.0001 compared to the control value.

As we had shown previously (Fig. 1 and reference 24), the S225A mutant NS5A exhibited a perinuclear restricted distribution. We therefore considered that the loss of interaction with cellular factors might be, at least in part, responsible for this restricted distribution. To test this hypothesis, we again used siRNA to ablate expression of NAP1L1, Bin1, VAP-A, or VAP-B in Huh7 cells stably harboring a wild-type SGR. Cells transfected with siRNA were analyzed by immunofluorescence with antibodies to both NS5A and the four cellular proteins. As shown in Fig. 7a, NAP1L1 ablation led to an overall reduction in NS5A expression which was restricted to a perinuclear distribution compared with the negative-control siRNA-transfected (scrambled) or untransfected (mock) cells. To provide quantitative confirmation of these observations, we determined NS5A spatial distribution data for 12 randomly selected cells (Fig. 7a, graph). A similar pattern was observed when Bin1 (Fig. 7b) or VAP-A (Fig. 8a) expression was ablated. In contrast, ablation of VAP-B expression had only a modest effect on the distribution of NS5A which did not reach significance upon quantitative analysis (Fig. 8b). Lastly, we sought to confirm that the effects of NAP1L1, Bin1, or VAP-A ablation on NS5A distribution were not due to a reduction in levels of expression, as an indirect consequence of the inhibition of genome replication. To test this, Huh7-Lunet T7 cells were transfected with pTM-NS3-5B followed by NAP1L1, Bin1, or VAP-A siRNAs. As shown in Fig. 9, the restricted distribution of NS5A was also observed using this expression system, confirming that the phenotype was not dependent on genome replication or the level of NS5A expression. These data demonstrate that S225 phosphorylation is required for the interaction of NS5A with a number of cellular proteins and that these interactions control both the distribution of NS5A and HCV genome replication.

## DISCUSSION

In this study, we used a proteomic approach to identify cellular proteins that interacted with NS5A in an S225 phosphorylation-dependent fashion, using affinity purification followed by liquid chromatography-tandem mass spectrometry (LC-MS/MS). We focused our attention on proteins that were enriched in samples derived from OST-tagged wild-type NS5A and that were significantly less abundant in samples from OST-tagged NS5A (S225A).

The most intriguing S225 phosphorylation-dependent interacting proteins were NAP1L1 and NAP1L4. These are members of the nucleosome assembly protein 1 (NAP1) family and, as their name suggests, are nuclear proteins primarily involved in chromatin assembly. However, they are also located in the cytoplasm, particularly during G_2_, and mediate nucleocytoplasmic shuttling of histones during cell cycle progression (40). Of the five mammalian NAP1 family members (NAP1L1 to NAP1L5), only NAP1L1 and NAP1L4 are ubiquitously expressed, while the other three are expressed mainly in neurons (41). NAPs have been shown to interact with proteins of a number of DNA viruses, including papillomavirus E2 (42), Epstein-Barr virus EBNA1 (43), and KSHV LANA (35). NAP1L1 also binds to HIV-1 Tat and Rev, and these interactions regulate viral infectivity and the subcellular localization of the virus proteins (36, 44). Interestingly, both NAP1L1 and NAP1L4 were previously shown to interact with both the HCV core protein (33) and NS5A (34) when the viral proteins were expressed individually in HEK293 cells, although in neither study were these interactions validated. In contrast, our data reveal that in the context of the NS3-NS5B replication complex within Huh7 cells, both NAP1L1 and NAP1L4 interact with NS5A. Focusing on NAP1L1 (due to a lack of available immunological reagents for NAP1L4), we further showed that these interactions contribute to virus genome replication and the distribution of replication complexes throughout the cytoplasm. It will be intriguing to understand the precise role of these proteins in HCV genome replication; in this regard, NAP1 has been shown to oligomerize (45) and NAPs function to transport histones, two attributes that may be important for their function during the HCV life cycle.

We also identified Bin1 as an S225 phosphorylation-dependent NS5A interactor. Bin1 contains an N-terminal BAR domain, a C-terminal SH3 domain, and a central clathrin and adaptor binding domain (14, 26). Like other BAR domain-containing proteins, Bin1 is involved in membrane curvature and trafficking. It had previously been shown to interact via its SH3 domain with a polyproline motif (PxxPxR) located in NS5A LCSII (26, 27), and it also interacts with similar motifs in other viral proteins, such as alphavirus nsP3 (46). As LCSII is distal to LCSI, this suggests that S225 phosphorylation might effect a conformational change in NS5A that occludes the SH3 domain binding polyproline motif; alternatively, multiple contacts between the two proteins may be required to establish a stable interaction. The physiological role of Bin1 in regulating membrane curvature is consistent with its recruitment by NS5A to sites of replication where it may be involved in the rearrangements necessary to form the MW. As it is also involved in membrane trafficking, this provides a potential explanation for the perinuclear restriction of NS5A S225A replication complexes.

Both VAP-A and VAP-B have previously been shown to bind both NS5A and NS5B (30, 39). Interestingly, in the context of the genotype 1b Con1 isolate, residues T2185 and K2187 (corresponding to NS5A residues 215 and 217) were shown to be required for the interaction (32). Mutations of these residues abolished the interaction with VAP-A, although this was demonstrated using the yeast two-hybrid system. An additional culture-adaptive mutation (S2201I, corresponding to S229), could rescue the interaction, although this mutation resulted in a loss of hyperphosphorylation. Together with our data, these results suggest that LCSI and proximal sequences at the C terminus of domain I are required for the interaction with VAP-A. Furthermore, it was recently reported that the NS5A interaction with VAP-A was indirect and required a bridging protein, G protein pathway suppressor 2 (GPS2) (29). VAP-A and VAP-B are integral membrane proteins that have a range of reported functions pertaining to vesicle trafficking and lipid transport (30, 47). VAP-A also interacts with the oxysterol binding protein (OSBP), which is recruited to the MW along with PI4K (48), and is reported to regulate cholesterol transport to the MW. Although OSBP was not identified in our proteomic study, these observations are consistent with a role for S225 phosphorylation in regulating MW formation and distribution via an interaction with VAP-A.

In conclusion, we propose that phosphorylation of NS5A at S225 within LCSI contributes to efficient genome replication by regulating interactions with key cellular factors, some of which we have identified and characterized in this study. It is important to note that multiple phosphorylation sites have been identified within LCSI, and it is conceivable therefore that the total pool of NS5A within a cell might contain many distinct phosphorylated species, each of which could also have a different function(s). Identifying these distinct NS5A species is a technical challenge which is limited by the sensitivity of mass spectrometric methods. In this context, we were previously able to unambiguously identify by mass spectrometry an NS5A species that was phosphorylated within LCSI only on S222 and S225 (18). The abundance of this species is consistent with a specific role during virus genome replication. Given the likely interdependence of different phosphorylation events, exemplified by recent evidence for hierarchical phosphorylation, i.e., the requirement for S235 to be phosphorylated prior to S238 phosphorylation (16), it will also be challenging to understand the functions of these different NS5A species. A complete understanding of either the complexity of NS5A phosphorylation or the functional consequences remains a distant and aspirational objective. By demonstrating the role of phosphorylation in regulating NS5A-host protein interactions, we believe that this study makes a significant contribution to this objective; however, it provides only a few pieces of the jigsaw puzzle! There is much yet to be discovered.

## MATERIALS AND METHODS

### HCV replicon constructs.

A DNA construct of the neomycin phosphotransferase (Neo) containing subgenomic replicon pSGR-Neo-JFH-1-5A-OST, in which the One-Strep tag (OST) was introduced into the C terminus of NS5A domain III (25), was used in this study (Fig. 1a and b). Previously, the OST has been shown to have no effect on virus genome replication (25). Wild-type pSGR-Neo(*Rsr*II^del^)-JFH-1-5A-OST was first constructed by abolishing an RsrII site within the Neo gene using a Q5 site-directed mutagenesis kit (New England BioLabs [NEB]). Then the desired NS5A mutants from SGR-luc-JFH-1 (24) were cloned into pSGR-Neo(*Rsr*II^del^)-JFH-1-5A-OST via flanking Nsil/RsrII, and correct insertion was confirmed by sequencing.

### Cell culture.

Huh7 cells were maintained in Dulbecco's modified Eagle's medium (DMEM; Sigma-Aldrich) supplemented with 10% fetal bovine serum (FBS), 100 IU of penicillin/ml, 100 μg of streptomycin/ml, and 1% nonessential amino acids (NEAA) in a humidified incubator at 37°C with 5% CO_2_. Huh7 cells carrying a subgenomic JFH-1 replicon (SGR-Neo-JFH-1) were maintained in the same medium supplemented with 300 μg/ml of G418 (BioPioneer). Huh7-Lunet T7 cells (expressing T7 RNA polymerase, a kind gift from Volker Lohmann) were maintained in the same medium supplemented with 5 μg/ml of Zeocin (Life Technologies).

### Electroporation of replicon RNA and generation of stable cell lines.

The preparation of *in vitro* transcripts and electroporations for pSGR-Neo-JFH-1-5A-OST-derived constructs were conducted as described previously (25). In brief, 2 × 10^6^ Huh7 cells in diethyl pyrocarbonate (DEPC)–phosphate-buffered saline (PBS) were electroporated with 3 μg of *in vitro* RNA transcripts using a square-wave protocol at 260 V for 25 ms. Subsequently, cells were resuspended in complete DMEM and seeded at a culture area of 1 × 10^4^ cells/cm^2^ into 10-cm dishes. Forty-eight hours postelectroporation, the cells were selected with 300 μg/ml of G418. After 2 weeks, emerging colonies were pooled and kept under continuous G418 selection for 1 to 2 weeks in order to establish stable SGR-harboring cell lines. The maintenance of the S225 mutations was verified by RT-PCR and sequencing.

### Affinity purification coupled to quantitative LC-MS/MS proteomics.

To detect proteins bound to OST-tagged NS5A by affinity purification and mass spectrometry, cell pellets from Huh7 cells stably harboring NS5A wild-type (WT) and S225A and S225D mutant SGRs were prepared by snap-freezing cells in liquid nitrogen. Cell pellets were lysed in TAP lysis buffer (50 mM Tris-HCl [pH 7.5], 100 mM NaCl, 5% [vol/vol] glycerol, 0.2% [vol/vol] Nonidet P-40, 1.5 mM MgCl_2_) in the presence of protease inhibitor cocktail (EDTA free, cOmplete; Roche), phosphatase inhibitor (PhosSTOP; Roche), and 750 U of Benzonase (Core Facility, Max Planck Institute of Biochemistry [MPI-B]) for 30 min on ice. After incubation on ice, cell lysates were sonicated using a Bioruptor with 15 alternating 30-s on/off cycles and clarified by centrifugation at 16,000 × *g*. Subsequently, Strep-Tactin Sepharose beads (Iba) were incubated with 2 mg of protein of clarified lysate for 60 min at 4°C and washed once with TAP lysis buffer and three times with TAP wash buffer (50 mM Tris-HCl [pH 7.5], 100 mM NaCl, 5% [vol/vol] glycerol, 1.5 mM MgCl_2_) lacking Nonidet P-40 to remove residual detergent. Sample preparation and LC-MS/MS analysis were performed as described previously (49). Briefly, four independent affinity purifications were performed for each bait, samples were sequentially digested with LysC (Wako Chemicals, USA) and trypsin (Promega), acidified with 0.1% trifluoroacetic acid (TFA), desalted with C18 stage tips, and analyzed by liquid chromatography coupled to mass spectrometry on an Orbitrap XL instrument (Thermo Fisher Scientific). Mass spectrometry raw files were processed with MaxQuant software version 1.5.5.1 (50) using the built-in Andromeda engine to search against human and JFH-1 proteomes containing forward and reverse sequences. Additionally, the label-free quantification (LFQ) (51) algorithm and Match Between Runs option were used. Perseus software version 1.5.5.1 was used to further process the data. In this manner, only proteins identified on the basis of at least two peptides and a minimum of three quantification events in at least one experimental group were considered. LFQ protein intensity values were log transformed and missing values filled by imputation. Significantly enriched proteins were determined by Welch's *t* test with permutation-based false-discovery rate (FDR) statistics, performing 250 permutations. The FDR threshold was set to 0.01 and S0 parameter was set to 0.1 to separate background from specifically enriched proteins. Results were plotted using R (https://www.R-project.org) and visually adapted using Adobe Illustrator.

### SDS-PAGE and Western blotting.

Cells were washed twice in PBS, lysed in 1× Glasgow lysis buffer (GLB; 1% [vol/vol] Triton X-100, 120 mM KCl, 30 mM NaCl, 5 mM MgCl_2_, 10% [vol/vol] glycerol, and 10 mM PIPES-NaOH, [pH 7.2], with protease and phosphatase inhibitors) and harvested by centrifugation (2,800 × *g*, 10 min, and 4°C) before determination and normalization of protein concentration by bicinchoninic acid (BCA) assay (Pierce). Following separation by SDS-PAGE, proteins were transferred to a polyvinylidene difluoride (PVDF) membrane and blocked in 50% (vol/vol) Odyssey blocking (OB) buffer (LI-COR) in Tris-buffered saline (TBS). The membrane was incubated with primary antibodies overnight at 4°C, followed by secondary antibodies for 2 h at room temperature, both prepared in 25% OB buffer. Primary antibodies used were anti-NS5A (sheep, prepared in-house) at 1:4,000 (52), anti-NAP1L1 (rabbit; Santa Cruz) at 1:350, anti-Bin1 (rabbit; Generon) at 1:300, anti-VAP-A (rabbit; Generon) at 1:1,000, anti-VAP-B (rabbit; Generon) at 1:1,000, and anti-α-actin (mouse; Sigma) at 1:10,000. Secondary antibodies were anti-rabbit, anti-sheep (800 nm), or anti-mouse (700 nm) antibodies, used at 1:10,000 prior to imaging using a LI-COR Odyssey Sa infrared imaging system. Quantification of Western blots was carried out using Image Studio v3.1 (LI-COR) using a background subtraction method.

### PLA.

Cells were washed with PBS before fixation for 15 min at room temperature in 4% (wt/vol) paraformaldehyde (PFA); cells were subsequently permeabilized in 0.1% (vol/vol) Triton X-100–PBS and blocked with PBS-Tween (PBS-T) and 5% (wt/vol) bovine serum albumin (BSA) before immunostaining for anti-NS5A (mouse monoclonal, 1:1,000) and either anti-NAP1L1 (rabbit monoclonal, 1:100) or anti-Bin1 (rabbit monoclonal, 1:50) overnight at 4°C. Coverslips were washed 3 times for 5 min in PBS-T buffer under gentle shaking and incubated with proximity ligation assay (PLA) probes Duolink In Situ PLA Probe Anti-Mouse PLUS (DUO92001; Sigma-Aldrich) and Duolink In Situ PLA Probe Anti-Rabbit MINUS (DUO92005) for 2 h at 37°C. For PLA, all incubations were performed in a preheated humidity chamber and according the manufacturer's recommendations using a Duolink In Situ Detection Reagents Red kit (DUO92008). Coverslips were washed 3 times for 5 min in PBS-T buffer under gentle shaking and incubated with a DNA ligase previously diluted in ligation buffer for 30 min at 37°C. Coverslips were washed 3 times for 5 min in PBS-T buffer under gentle shaking and incubated with a DNA polymerase previously diluted in amplification buffer for 90 min at 37°C. Finally, coverslips were washed for 20 min under gentle shaking and then washed for 2 min with PBS and air dried. Coverslips were mounted with Duolink In Situ mounting medium with 4′,6-diamidino-2-phenylindole (DAPI), and fluorescence was visualized with a Zeiss LSM880 upright microscope.

### siRNA ablation.

SGR-harboring Huh7 cells or Huh7-Lunet T7 cells were transfected with 10 nM pooled siRNA (Santa Cruz) or 10 nM AllStars negative-control siRNA (Qiagen) using Lipofectamine 2000 (Invitrogen) according to the manufacturer's protocol. sc-75871 targets NAP1L1, sc-29804 targets Bin1, sc-61768 targets VAP-A, and sc-61770 targets VAP-B. For Huh7-Lunet T7 cell experiments, the cells were transfected with pTM-NS3-5B plasmid at 48 h posttransfection (hpt). Cells were incubated in DMEM supplemented with 5% fetal calf serum (FCS) and harvested at 72 hpt. Proteins and total RNA were isolated with TRIzol (Life Technologies), and subsequent Western blotting and real-time quantitative PCR (qRT-PCR) were performed.

### RNA extraction and qRT-PCR.

To quantify the number of HCV genomes, total cell RNA was extracted using TRIzol reagent by following the manufacturer's instructions (Invitrogen). Total extracted cellular RNA (100 ng) was analyzed using a one-step qRT-PCR TaqMan-based kit (Eurogentec), with primers and probe designed against the 5′ untranslated region (UTR) as described previously (19, 53).

### Immunofluorescence and confocal microscopy.

Cells were washed with PBS before fixation for 20 min in 4% (wt/vol) PFA; cells were subsequently permeabilized in 0.1% (vol/vol) Triton X-100 and PBS and blocked with PBS-T and 5% (wt/vol) BSA before being immunostained with primary antibody as described above. Various fluorescently conjugated secondary antibodies were used at 1:500 (Life Technology). Nuclei were counterstained with DAPI. Confocal microscopy images were acquired on a Zeiss LSM880 upright microscope with Airyscan; postacquisition analysis was conducted using Zen software (Zen version 2015 black edition 2.3; Zeiss) or Fiji (version 1.49) software (54).

### dSTORM.

The direct stochastic optical reconstruction microscopy (dSTORM) system described previously (55) was modified with a cylindrical lens (*f* = 150 mm, where *f* is focal length; Thorlabs). Round glass 25-mm-diameter coverslips (Warner Instruments) were cleaned in a 1:1:5 solution of NH_3_ (aq), H_2_O_2_, and H_2_O at 80°C for 16 h. Cells stably harboring NS5A wild-type, S225D, and S225A mutant subgenomic replicons were seeded onto cleaned coverslips at 1 × 10^5^ cells per well in six-well plates. Coverslips were fixed in 2% (wt/vol) PFA in normal medium for 10 min, permeabilized in 0.2% Triton X-100 in PBS for 10 min, and blocked in 1% (vol/vol) normal donkey serum (Sigma-Aldrich) for 1 h. Mouse monoclonal anti-NS5A antibody was directly labeled at an approximately 1:1 ratio using the carboxylic acid succinimidyl ester of the photoswitchable dye Alexa Fluor 647 (Life Technologies) in PBS containing 125 mM NaHCO_3_ in the dark for 30 min. Unincorporated dye was removed by size exclusion using Zeba microspin desalting columns (Thermo Fisher Scientific). Immunostaining was conducted with directly labeled antibody (1:2,000) for 1 h. Coverslips were then treated with 0.01% poly-l-lysine (Sigma-Aldrich) for 10 min and incubated with a suspension of 150-nm gold nanoparticles (Sigma-Aldrich).

The image acquisition and processing software was used as described previously (56). Labels were stochastically activated with 642-nm laser excitation under wide-field illumination in the presence of fluorescence quenching buffer (glucose oxidase [10 U], catalase [50 U], 12.5 mg ml^−1^ of d-glucose, 1 mM 2-mercaptoethylamine in PBS [pH 8.0]). Data sets consisted of 11,000 image frames at a frame rate of 20 Hz. Localized emission events were binned into histograms for display and correction of image distortion by the cylindrical lens. Image smoothing was conducted in R using kernel density estimation to reflect *x-y-z* localization precisions measured from fiducial markers, and *z*-stacks were visualized in Fiji. Clustering analysis was conducted in Python using density-based spatial clustering of applications with noise (DBSCAN) (57) on the localization coordinates extracted from the palm3d software (MinPts = 30; ε = 150 nm). Cluster sizes were determined from the mean Euclidean distance between all localizations in identified clusters. Image analysis in R and Python used custom scripts (available on request), and statistical tests were conducted in GraphPad Prism using a one-way analysis of variance (ANOVA) with Tukey's multiple comparisons.

### Quantification of NS5A distribution.

For quantification of NS5A spatial arrangement, images were acquired with the same acquisition parameters, but with variable gain to ensure correct exposure. The spatial coordinates of NS5A were determined using the FindFoci function of the GDSC plugin for Fiji, with the nuclear envelope being manually outlined (utilizing the DAPI staining as a reference) and coordinates generated by Fiji. The distance from each NS5A to the nuclear envelope was then determined using trigonometry. NS5A spatial distribution data were generated for 12 randomly selected cells for each replicon variant and data combined into a box-and-whisker plot.

For colocalization analysis, Manders' overlap coefficient was calculated using ImageJ software with Just Another Co-localization Plugin (JACoP) (National Institutes of Health). Coefficient M1 reflects the fraction of the anti-NS5A signal that overlaps either the anti-NAP1L1 or anti-Bin1 signal. Coefficient M2 reflects the fraction of either the anti-NAP1L1 or anti-Bin1 signal that overlaps the anti-NS5A signal. Coefficient values range from 0 to 1, corresponding to nonoverlapping images and 100% colocalization images, respectively. Colocalization calculations were performed on >10 cells from at least two independent experiments.

### Statistics.

Data sets were analyzed using Student's *t* test assuming a two-tailed, unequal variance to determine statistical difference from the wild type (WT) (*n* = 3 or greater throughout).

## Supplementary Material

Supplemental material
