# Supplementary material for: Phosphorylation of Serine 225 in Hepatitis C Virus NS5A Regulates Protein-Protein Interactions
Source: J Virol. 2017 Aug 10;91(17):e00805-17. doi: 10.1128/JVI.00805-17 (PMC5553161; doi:10.1128/JVI.00805-17)
Supplement: Supplemental material [file supp_91_17_e00805-17__index.html]

Phosphorylation of Serine 225 in Hepatitis C Virus NS5A Regulates Protein-Protein Interactions — Supplemental material 

# Phosphorylation of Serine 225 in Hepatitis C Virus NS5A Regulates Protein-Protein Interactions

## Supplemental material

- Supplemental file 1 -

  Data Set S1 (Proteomic data.)

  XLSX, 62K
